# Autophagy in Measles Virus Infection

**DOI:** 10.3390/v9120359

**Published:** 2017-11-24

**Authors:** Aurore Rozières, Christophe Viret, Mathias Faure

**Affiliations:** 1 International Center for Infectiology Research (CIRI), Université de Lyon, 69007 Lyon, France; christophe.viret@inserm.fr (C.V.); mathias.faure@inserm.fr (M.F.); 2 Inserm, U1111, 69007 Lyon, France; 3 CNRS, UMR5308, 69007 Lyon, France; 4 Ecole Normale Supérieure de Lyon, 69007 Lyon, France; 5 Université Lyon 1, Centre International de Recherche en Infectiologie, 69007 Lyon, France; 6 Equipe FRM Labellisée Fondation Pour la Recherche Médicale FRM, 75007 Paris, France

**Keywords:** measles virus, autophagy, selective autophagy, autophagy receptors, CD46, IRGM, NDP52, T6BP, OPTN

## Abstract

Autophagy is a biological process that helps cells to recycle obsolete cellular components and which greatly contributes to maintaining cellular integrity in response to environmental stress factors. Autophagy is also among the first lines of cellular defense against invading microorganisms, including viruses. The autophagic destruction of invading pathogens, a process referred to as xenophagy, involves cytosolic autophagy receptors, such as p62/SQSTM1 (Sequestosome 1) or NDP52/CALCOCO2 (Nuclear Dot 52 KDa Protein/Calcium Binding And Coiled-Coil Domain 2), which bind to microbial components and target them towards growing autophagosomes for degradation. However, most, if not all, infectious viruses have evolved molecular tricks to escape from xenophagy. Many viruses even use autophagy, part of the autophagy pathway or some autophagy-associated proteins, to improve their infectious potential. In this regard, the measles virus, responsible for epidemic measles, has a unique interface with autophagy as the virus can induce multiple rounds of autophagy in the course of infection. These successive waves of autophagy result from distinct molecular pathways and seem associated with anti- and/or pro-measles virus consequences. In this review, we describe what the autophagy–measles virus interplay has taught us about both the biology of the virus and the mechanistic orchestration of autophagy.

## 1. Introduction

Measles virus (MeV) is a member of the *Morbillivirus* genus in the *Paramyxoviridae* family responsible for Measles, a common childhood infectious disease characterized by high fever, respiratory infection and typical macullo papular rash [1]. This virus first affects the respiratory tract before disseminating within the whole body. An adaptive immune response follows and consists of both cellular and humoral responses responsible for the establishment of long-term protective immunity. MeV infection is associated with a profound immunosuppression occurring 10–14 days post-infection, which is associated with secondary opportunistic infections and, in a fraction of cases, post-infectious subacute sclerosing panencephalitis (SSPE) with a high rate of morbidity and mortality [2]. Despite effective vaccines, MeV still causes millions of infections worldwide every year [1].

MeV is an enveloped virus with a non-segmented negative strand RNA genome, which codes for six structural proteins (N, P, L, H, F, and M) and two non-structural proteins (V and C). The two envelope glycoproteins, the hemagglutinin (H) and fusion (F) proteins, play a crucial role in receptor-binding and membrane fusion. MeV entry requires membrane fusion and, to date, three different host–cell receptors have been described as entry receptors. Clinical/virulent strains of MeV invade, first, the immune cells using CD150/SLAM (Signaling Lymphocytic Activation Molecule) expressed on dendritic cells and macrophages. Then, clinical/virulent strains invade epithelial cells by binding to Nectin 4 expressed on their basolateral surface [3,4,5]. Attenuated/vaccinal MeV strains bind to an additional receptor, CD46, a complement regulatory molecule, which is expressed on all nucleated somatic cells [6]. H/receptor interaction and engagement of F induce conformational modification of the F protein leading to membrane fusion and virus genome delivery within the infected cell. The virus replicates in the cytosol and spreads by budding from the cell surface of infected cells. MeV infection can also lead to the formation of syncytia, as the result of cell–cell fusion between infected cells and uninfected cells [7].

**Figure 1:**
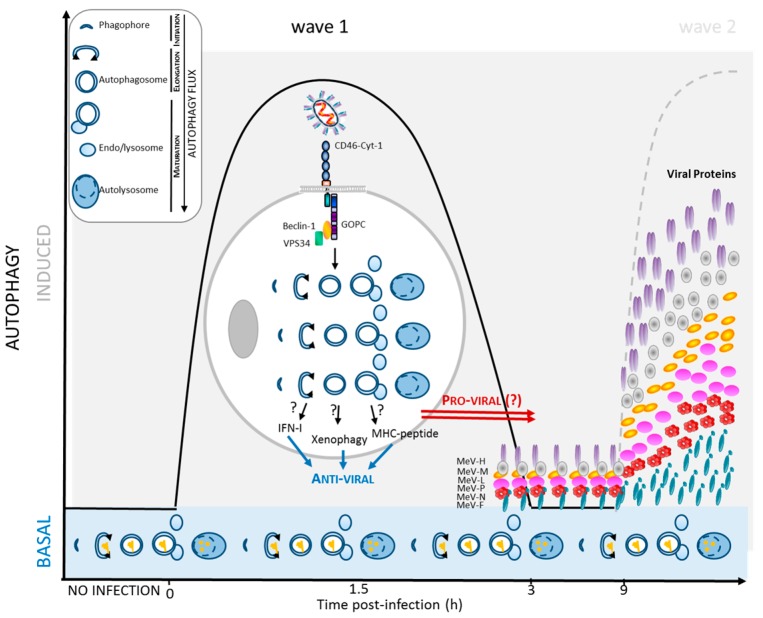
Early induction of autophagy upon MeV infection. Attenuated MeV strains induce autophagy as soon as 1.5 h after infection via a CD46/GOPC (Golgi Associated PDZ and Coiled-Coil Motif Containing)-dependent pathway. This wave of autophagy could contribute to protecting the infected cells and surrounding cells from further infections due to its anti-viral potential. On the other hand, infectious MeV could use such an autophagy induction to facilitate its own replication (pro-viral). Before induction of a second wave of autophagy, which correlates with the accumulation of viral proteins, the first transient wave of autophagy ceases. Basal autophagy remains possibly efficient during the all cycles of MeV infection. Note that virulent/clinical strains of MeV, which do not bind to CD46, do not induce such an early autophagy wave. Upper left panel: schematic representation of the main steps of the complete autophagy flux process. (blue arrows = anti-viral effect; red arrows = pro-viral effect; dotted line = wave 2).

Macroautophagy, hereafter referred to as autophagy, is an intracellular degradation system required for cell homeostasis. It allows for the constant degradation and recycling of cytoplasmic components at a steady state, and is modulated in response to changes in the cellular microenvironment. The process is a lysosomal-dependent mechanism initiated by the formation of an isolated membrane, the phagophore, which elongates and entraps cytosolic components to be degraded within a newly-formed vesicle called an autophagosome. Upon fusion with endo/lysosomes, autophagosomes form autolysosomes in which degradation occurs (the upper left panel in Figure 1). The unperturbed occurrence of these three ordered main steps (initiation of an isolated membrane, elongation of both extremities of the phagophore, and maturation of the autophagosome by fusion with a lysosome corresponding to the degradation step) constitutes the so-called autophagy flux. Execution of autophagy and regulation of the autophagy machinery rely on autophagy-related (ATG) proteins and dozens of additional contributing proteins in mammalian cells [8].

In addition to regulating cell homeostasis, the autophagy machinery can also be used as a cell defense mechanism against intracellular microbes, such as bacteria or viruses, through their degradation (a process referred to as xenophagy) [9,10]. In addition, autophagy contributes to modulating innate immunity through the intracellular recognition and targeting of pathogens to autophagosomes, but also through positive or negative regulation of pattern recognition receptor (PRR) and damage-associated molecular pattern (DAMP) receptor signaling [11]. Autophagy can also contribute to adaptive immune responses by delivering peptides for presentation by major histocompatibility complex (MHC) molecules on antigen-presenting cells to T lymphocytes [12]. Due to the crucial roles of the autophagy process in the context of infections and immunity, measles virus–autophagy interplay has been recently investigated, revealing an intricate cross-talk that is reviewed thereafter.

## 2. Autophagy Induction in MeV Infection

### 2.1. Autophagy Detection upon MeV Infection

**Figure 2:**
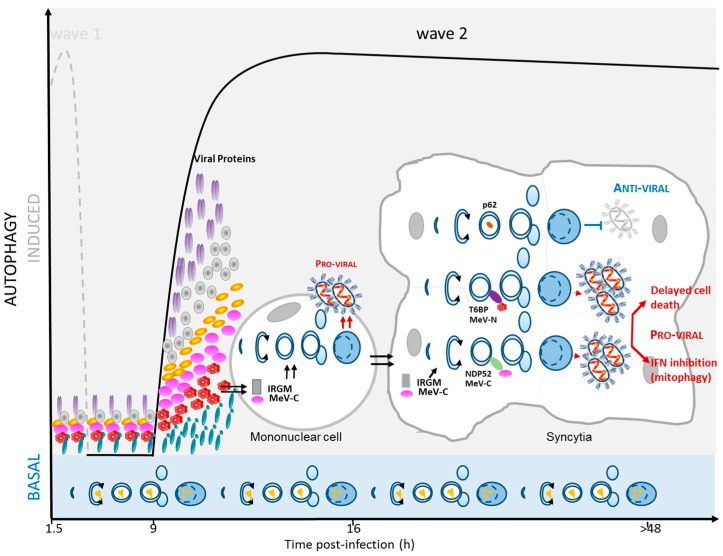
Second wave of autophagy induction upon MeV infection. After nine hours of MeV infection, a second autophagy wave is induced, which correlates with the accumulation of MeV proteins. Several successive signals seem associated with this late autophagy wave, including IRGM engagement and events associated with syncytia formation. Autophagosome maturation is required for an efficient MeV replication (pro-viral effect). This effect could involve the two autophagy adaptors NDP52 (Nuclear Dot 52 KDa Protein) and T6BP (TRAF6-Binding Protein), which are both targeted by MeV proteins. In contrast, p62 appears to oppose MeV replication (anti-viral effect). The pro-viral function of autophagy has been shown to involve a delayed death of infected cells and a decreased innate anti-viral response. (blue arrows = anti-viral effect; red arrows = pro-viral effect; dotted line = wave 1).

Many, if not all, infectious pathogens have to deal with the autophagy machinery upon cell invasion. Among those pathogens, cell infection with MeV was shown to correlate with the accumulation of autophagosomes [13,14,15,16]. Strikingly, the kinetics of autophagy induction upon MeV infection revealed two successive waves of autophagy [13,16]. The first wave of autophagy induction was observed very early post MeV entry, as soon as 1.5 h post infection [13,15] (Figure 1). This early induction is observed only upon attenuated/vaccinal MeV strain infection (not with virulent strains), and is very transient since the basal level of autophagy was recovered by three hours after infection [13]. Interestingly, this basal level corresponds to a complete functional autophagy flux, suggesting that the decrease of flux intensity, at this point, is not due to strategies evolved by attenuated/vaccinal MeV to inhibit autophagy, but to a termination of signaling (Figure 1). Subsequently, a second wave of autophagy was induced nine hours post-infection by MeV [13,14] (Figure 2). This autophagy signal is observed upon infection with both virulent and attenuated/vaccinal strains of MeV [13]. Moreover, this second autophagy wave persists for a very long period of time since, up to 48 h post MeV infection, the number of autophagosomes was found to still be far higher in infected cells than in non-infected cells [13,14]. The occurrence of two successive waves, one early and one late, suggests that distinct molecular mechanisms are involved in the induction of autophagy in the course of MeV infection.

### 2.2. MeV Entry and Early Autophagy Induction

The very early wave of autophagy induction upon attenuated/vaccinal MeV infection suggests that virus entry might signal for triggering such a process. Indeed, newly-synthesized viral proteins were not required for the induction of the early autophagy wave, since its occurrence was not altered by cycloheximide and non-replicative, UV-treated MeV still induced this wave [13]. Pioneering works in our laboratory described the first attenuated/vaccinal MeV receptor as being CD46 [6]. This ubiquitously-expressed receptor is known as a member of the receptor complement activation (RCA) family as its function is to protect nucleated human cells from complement-mediated autolysis [17]. CD46 has the unique property to co-stimulate naïve human T cells upon T cell receptor engagement, leading to the differentiation of regulatory Tr1-like T cells [18,19]. Among the 12 isoforms of CD46 that can be expressed, only two different cytoplasmic tails are found [20]. The so-called intracellular portions Cyt-1 and Cyt-2 are completely different in sequence and were described to be associated with distinct intracellular trafficking [21]. Interestingly, it was found that the absence of CD46-Cyt-1 prevents early autophagy induction by attenuated/vaccinal MeV infection [15]. Moreover, the unique engagement of CD46-Cyt-1 with crosslinking antibodies induces the formation of autophagic vesicles. Furthermore, it was reported that the Cyt-1 intracytoplasmic tail can associate with the scaffold protein GOPC, which connects CD46-Cyt-1 to the autophagy machinery by interacting with the autophagosome formation complex BECN1(Beclin 1)-VPS34(Vacuolar Protein Sorting 34) [22]. The absence of GOPC did not modulate the membrane expression of CD46-Cyt-1, but prevented attenuated/vaccinal MeV infection-mediated early autophagy induction in infected cells [15]. Thus, attenuated/vaccinal MeV infection revealed a specific pathway for autophagy induction, involving CD46-Cyt-1 (but not CD46-Cyt-2) and GOPC for initiation of autophagosome formation via BECN1 interaction. How precisely such a molecular mechanism is orchestrated in the course of infection remains unknown. However, this specific pathway is only used by attenuated/vaccinal MeV, and not by virulent MeV, which do not bind to CD46 to infect cells. A virulent strain of MeV, which uses CD150/SLAM to infect cells, was found unable to induce early autophagy upon entry [13]. This suggests that no other signaling pathways, such as those emanating from PRR engagement, are triggered upon MeV entry to induce autophagy [23]. However, whether virulent strains use CD150/SLAM for not inducing autophagy, or whether they have evolved mechanisms to block early autophagy signaling is unknown.

### 2.3. MeV Replication and Late Autophagy Induction

Following the CD46-dependant autophagy induction, a transient autophagic steady state is retrieved within infected cells. At this step, autophagy was perfectly functional in infected cells (Figure 1). However, a few hours after infection, a second wave of autophagy induction was observed [13] (Figure 2). This late wave correlated in time with the exponential accumulation of viral transcripts and with the accumulation of newly-synthesized viral proteins [24]. Moreover, newly-synthesized viral proteins were found to be absolutely required for the induction of the late autophagy wave, and non-replicative UV-treated MeV was found unable to induce this wave [13].

Viruses were found to massively interact with specific pathways involved in the usual control of infections [25]. The autophagy-related proteome is one of the major targets of RNA viruses [14]. This might reflect the importance of autophagy in the course of viral infections. This process could counteract infections (through the xenophagy process) or be used by pathogens (for instance, by supplying viruses with metabolites). Several MeV proteins were reported to be able to interact with several distinct autophagy-associated proteins, which could contribute to induce/modulate the late wave of autophagy: GOPC (with C), IRGM (Immunity Related GTPase M) (with C and N), p62 and UVRAG (UV Radiation Resistance-Associated Gene Protein) (both with N), NDP52 (with C and V), and T6BP (with N) [14,26,27].

Among such interactions, the putative targeting of GOPC by the MeV-protein MeV-C was reported to not impact the late phase of autophagy induction upon infection and, so, the exact role of this targeting in autophagy modulation is unclear [13]. Possibly, such an interaction could limit the role of GOPC in the induction of autophagy by potentially newly-entering viruses in already-infected cells.

The human immunity-related GTPase family M (IRGM) protein was shown to be targeted by at least two MeV proteins, C and N. IRGM was described to regulate autophagy mainly in the context of infection, suggesting a pathogen-specific function in autophagy for this protein. In human cells, IRGM-mediated autophagy contributes to controlling infection by intracellular bacteria: *M. tuberculosis, Escherichia coli*, and *Salmonella typhymurium* [28,29,30,31]. However, in the context of viral infection, several RNA viruses that manipulate autophagy to efficiently replicate were found to target IRGM [14]. The late autophagy wave induced upon MeV infection was found to be dependent on IRGM expression [14]. The overexpression of C protein, which interacts with IRGM, was found to be sufficient to induce autophagy via an IRGM-dependent pathway (Figure 2). Furthermore, an MeV strain defective for the expression of the C protein (MeVΔC) was found unable to efficiently induce the late IRGM-dependent wave of autophagy, although it was still efficient at inducing the early, CD46-dependant, autophagy wave [13]. How the C–IRGM interaction leads to autophagy induction remains unclear. IRGM can interact with several autophagy-associated proteins involved in the initiation of the autophagy process: ATG5, ATG10, LC3C (Light Chain 3 C), SH3GLB1 (SH3 Domain Containing GRB2 Like, Endophilin B1), ULK1 (Unc-51 Like Autophagy Activating Kinase 1), and BECN1, which could contribute to the induction of autophagy upon MeV-C expression [14,32,33]. The interaction of IRGM with ULK1 and BECN1 was also reported to be crucial in order to initiate the formation of autophagosomal membranes [32]. Strikingly, several other RNA viruses (HCV, HIV-1, Japanese encephalitis virus, Coxsackievirus A16) were found to require IRGM to efficiently induce autophagy upon infection, via potential interactions with one of their viral proteins (NEF, NS3, NS3, 2C, respectively) [14,34,35,36]. Upon HCV infection, IRGM is crucial to inducing the phosphorylation of ULK1, a key step to initiating autophagy [35]. A similar mechanism might apply to MeV infection, as well as to infections by other RNA viruses. Interaction of MeV with UVRAG has not yet been investigated, but could contribute to the late autophagy induction upon infection, since UVRAG is one of the molecular switches that can promote autophagosome formation [37].

### 2.4. MeV Replication and Very Late Autophagy Induction

The second (late) wave of autophagy is maintained at a high level in MeV-infected cells for a long period of time (>48 h post-infection). This sustained autophagy involves, on one hand, the maintained expression of MeV-C in infected cells and, on the other hand, the fusion events between infected cells and uninfected cells [13,38] (Figure 2). Indeed, the mechanical fusion of cells using PEG (polyéthylène glycol) or fusogenic viral proteins is correlated with an increase of autophagy in such induced multinucleated cells, suggesting that syncytia formation by itself enhances autophagy [13]. Numerous enveloped viruses, including MeV, can lead to the formation of syncytia. This could reflect the constant requirement of autophagy in this type of cells to efficiently recycle their own components in order to maintain-homeostasis, but the precise role of intense autophagy in multinucleated cell biology remains unknown.

Moreover, the treatment of MeV infected cells with a fusion inhibitory peptide (blocking the formation of syncytia) [39] did not prevent late autophagy induction. Furthermore, GOPC did not play a role in this late event, suggesting an involvement for CD46 in mediating cell–cell fusion, but not in inducing late autophagy (at least via GOPC) [13]. Thus, through the generation of syncytia, MeV infection also maintains a long lasting autophagy level. The molecular pathway(s) involved in this cell–cell fusion phenomenon is unknown, but it could be hypothesized that each time a newly-uninfected cell fuses with the growing syncytia, autophagy is stimulated. Furthermore, a positive loop for autophagy maintenance would favor the whole process that benefits MeV, with autophagy promoting virus production, which, in turn, favors cell–cell fusion, which further triggers autophagy.

Similar to many viruses, MeV itself appears resistant to cellular microRNAs [40]. However, whether the above described waves of autophagy induction by MeV also involve a role for microRNAs is unknown. This is a possibility since an increasing corpus of observation indicates that the autophagy machinery is highly susceptible to microRNA-mediated modulation [41,42] and particularly at the level of the crosstalk between autophagy and apoptosis [43].

## 3. Autophagy as a Pro-MeV Mechanism

### 3.1. Autophagy Induction Promotes MeV Particle Production

MeV infection with attenuated/vaccinal strains thus induces a strong autophagy flux with two successive waves (early and late). In contrast, virulent strains were only reported to induce the late wave of autophagy. It was found that initiation of the autophagic process is required for an efficient production of new viral particles; the reduced expression of ATG proteins limits the production of viral particles [13]. Thus, autophagy seems mainly to be used by MeV to facilitate its replication. In ATG-defective cells, both waves of autophagy are prevented and MeV replicates poorly. It was shown that MeV replication is compromised in the absence of IRGM, which is only responsible for the late MeV-induced autophagy induction [14]. This suggests that MeV, and potentially other RNA viruses, evolved to induce/hijack autophagy via the manipulation of a specific anti-microbial pathway. However, the exclusive role of IRGM in anti-microbial autophagy remains incompletely established, since some studies also reported a role for this protein in steady state cellular autophagy [28]. Nevertheless, blocking the induction of the late wave of autophagy compromised MeV replication, regardless of whether cells were infected with attenuated/vaccinal or virulent strains. Furthermore, promotion of MeV replication was also observed in infected cells with experimentally-enhanced levels of autophagy. Thus, MeV infection of cells pre-treated with rapamycin, a potent inducer of autophagy, leads to increased MeV particle production [13]. Most of these observations on the pro-viral role of autophagy induction were made in models of immortalized epithelial cell lines, and it would be important to determine whether similar events happen in primary human cells.

### 3.2. Mitophagy Promotes MeV Replication

Mitophagy is the selective degradation of mitochondria by the autophagic pathway [44]. It has been shown that upon attenuated MeV strain infection, mitochondria co-localize with LC3 over time, and some mitochondria were found within autophagosomal structures by electron microscopy. Thus, among autophagy signals induced upon MeV infection, certain could favor mitophagy [16]. Interestingly, MeV-induced autophagy flux was reported to impair anti-MeV innate immune responses. Among antiviral innate actors, newly-synthesized type I interferon (IFN-I) are very powerful cytokines of the innate immune response against viral infections. They bind to the IFN-I receptor (IFNAR), which transduces signals leading to the expression of hundreds of IFN stimulating genes (ISGs) that have a direct antiviral effect. In the context of MeV infection, defective autophagy resulted in improved synthesis of ISGs with anti-viral potency, such as IFN-β, IFI27, OAS1 (2′-5′-Oligoadenylate Synthetase 1), and CXCL10 (C-X-C Motif Chemokine Ligand 10) [16]. To modulate innate response, MeV-induced autophagy seems to attenuate the MAVS-related anti-viral signaling due to the physical degradation of MAVS-containing mitochondria by mitophagy. Upon MeV-infection, mitophagy, which reduces the overall mass of mitochondria in MeV-infected cells, was found to involve the autophagy receptor p62 [16]. As reported for another member of the Paramyxoviridae family, another pathway for mitophagy induction could rely on the expression of MeV-M since M protein of the human parainfluenza virus 3 (HPIV3) has been reported recently to mediate mitophagy through its interaction with the mitochondrial factor TUFM (Tu Translation Elongation Factor, Mitochondrial) and with LC3 [45].

### 3.3. Autophagy Maturation Is Required for Efficient MeV Particle Production

A striking observation in the context of MeV infection is that this virus induces a complete and productive autophagy flux (that is, from the initiation step to the maturation/degradation step). This is in contrast, for instance, with what has been observed in the context of the paramyxovirus HPIV3 (Human parainfluenza virus type 3) infection. HPIV3 blocks the SNAP29-STX17 (Synaptosome Associated Protein 29—Syntaxin 17)-mediated autophagosome maturation via the HPIV3-P protein leading to improved viral release [45,46]. Studies revealed indeed that cellular autophagy substrates are efficiently degraded by early and late MeV-induced autophagy. In contrast, viral proteins seem not to be massively targeted for autophagy degradation [13]. How viral components escape from this intense autophagic activity remains to be appreciated. It could result from an equilibrium between intense viral protein synthesis and degradation by autophagy, in favor of the virus. Nevertheless, expression of H and N transcripts were found to require functional autophagy, after 48 h of infection [16]. It is possible that the excess of viral transcripts is targeted to autophagic degradation, whereas viral proteins mostly escape from autophagic degradation. Autophagy maturation is a step absolutely required for efficient MeV replication. Indeed, treatment of cells with chloroquine, an inhibitor of autophagosome recycling, was found to limit the production of new MeV particles, demonstrating that the efficient production of MeV particles not only involve the neo-formation of autophagosomes, but also the full maturation of the formed autophagosomes [13]. It is possible that such a complete flux of autophagy is required to supply MeV in various recycled metabolites for its optimal replication. Another consequence of this intense and productive autophagy was found to be a delay in MeV-infected cell apoptosis [16,47]. Due to this delay, MeV could take advantage of an extended time to replicate and to assemble, increasing the production of new particles before cell death occurrence. Whether MeV-induced autophagy could possibly degrade apoptotic factors to delay the apoptosis of infected cells is unknown.

Among autophagy-associated proteins found to interact with MeV proteins, two of them were recently reported to regulate the maturation of autophagosomes: NDP52 and T6BP [26,48,49]. NDP52 is a known autophagic receptor, whose main function is to bind substrates and to target them towards growing autophagosomal membranes for their ultimate autophagic degradation. Recently, NDP52 was described to contain a domain markedly related to the LC3-interacting region (LIR) and which allows its interaction with several members of the LC3 family; LC3A, LC3B, and GABARAPL2 (GABA Type A Receptor Associated Protein Like 2) [48,50,51]. Furthermore, NDP52 contains a domain allowing its interaction with MYOSIN VI, a unique non-conventional myosin motor that travels towards the minus end of actin filaments, and which binds to the endosomal protein TOM1 (Target Of Myb1 Membrane Trafficking Protein) [52]. In this way, NDP52 facilitates the proximity of autophagosomes with vesicles of the endo/lysosomal pathway, which is required for the final step of autophagosome maturation [48]. Thus, NDP52 has a dual function during autophagy: while it serves as an autophagy receptor, it also serves as an autophagy adaptor to regulate the fusion of autophagosomes with compartments of the endo/lysosomal pathway. Similarly, OPTN (Optineurin) and T6BP, two other autophagy receptors, were observed to regulate autophagosome maturation [48]. Strikingly, it was observed that the absence of either NDP52 or T6BP compromises the efficient replication of MeV [26]. The opposite would have been expected if we consider the role of these two proteins as playing the role of an autophagy receptor to control MeV infection. Instead, MeV proteins could target these two proteins to facilitate autophagosome maturation, and improve MeV infectivity. Importantly, OPTN plays no obvious role in MeV replication, suggesting that the selective targeting of autophagy adaptors by MeV might have evolved to optimize viral replication.

Increasing evidence reveals that autophagy-associated proteins can be involved in the virulence of infectious viruses. For instance, autophagy proteins could be used to facilitate exocytosis of viruses from infected cells [53,54]. Whether such an autophagy protein hijacking is also exploited by MeV remains to be determined.

## 4. Autophagy as an Anti-MeV Cellular Mechanism

### 4.1. Autophagy Contribution to Anti-MeV Immune Responses

As mentioned above, while the late wave of autophagy is induced by both attenuated/vaccinal and virulent strains of MeV, the early one is only induced upon attenuated/vaccinal MeV strain entry through a specific CD46-Cyt-1/GOPC pathway. Although transient, this early phase of autophagy induction could possibly be used by infected cells to resist invading pathogens through the degradation of some viral components and/or by promoting antiviral innate and adaptive immune responses

### 4.2. Induction of Intrinsic and Innate Anti-Viral Responses

Xenophagy of recently-entered MeV particles could be an efficient way to limit infectivity prior to viral replication. MeV particles were not observed in CD46-induced autophagosomes, but, to date, few viral particles were described to be effective targets of autophagy [10,55]. However, individual viral components could be degraded to limit replication, as observed for Sindbis virus or Chikungunya virus [56,57]. We could suppose that, upon infection, depending on the ratio of pathogens versus cells, MeV xenophagy could be sufficient to overcome productive infection. 

In line with this hypothesis, among autophagy-associated proteins found to interact with MeV proteins, is the autophagy receptor p62 (with N) [26]. It was found that the reduced expression of p62 leads to a more efficient production of viral particles. Thus, unlike NDP52 and T6BP, whose functions in MeV replication seem mainly associated with the regulation of autophagy maturation, p62 could solely play the role of the autophagy receptor in order to target viral components for viral xenophagy. The interaction of p62 with N could, therefore, be associated with partial autophagic degradation of N, limiting the replication of MeV. Although this could happen as soon as N-associated genome delivery within the cell following MeV entry, we might expect this process more efficient in the late phase of autophagy, during which N is accumulating (Figure 2).

In addition to xenophagy, autophagy can serve to target viral genomes to endosomes containing viral-nucleic acid specific Toll-like receptors (TLRs), which are membrane-expressed innate receptors, following the fusion of viral genome-containing autophagosome with endosomes (in the so-called amphisomes) [58]. Thus, TRL7 (Toll Like Receptor 7), located within endosomal compartments, can recognize viral genomic molecular determinants entrapped by autophagosomal membranes [58]. TLR7 signaling leads to the activation of IFN-I regulatory transcription factors (IRF), for IFN-I synthesis. Early CD46-induced autophagy could, thus, be solicited to capture and concentrate the viral genome delivered within the cytosol, in order to promote anti-viral IFN-I synthesis, which would not be the case upon virulent strains’ infection. Whether early autophagy contributes to such an IFN-I production, however, remains unknown.

Among open questions is also the issue of whether autophagy, or the autophagy machinery, can influence the production of type III interferon that can be seen in epithelial cells upon MeV infection [59].

### 4.3. Induction of Anti-Viral Adaptive Immune Response

Autophagy has also been described to contribute to endogenous and exogenous-derived peptide presentation by class II major histocompatibility complex (MHC) molecules in antigen presenting cells. Autophagosomes could fuse with MHC-II rich vesicles to deliver degraded peptides on MHC-II molecules for presentation to specific CD4^+^ T cells [53,54,60,61]. Thus, CD46-mediated autophagy could supply MeV-infected antigen presenting cells with antigenic MeV-derived peptides. This would contribute to an early and efficient initiation of an anti-viral adaptive immune T cell response by stimulating MeV-derived peptide-specific naïve CD4^+^ T cells. Supporting this hypothesis, it has been reported that CD46 contributes to MeV-derived peptide presentation on MHC-II molecules via a vesicular pathway that is sensitive to chloroquine, which involves the endosomal compartment [62,63]. In particular, the neo-expression of CD46 by mouse B lymphocytes led to the efficient presentation of MeV-H- and MeV-N-derived peptides by MHC-II to T cell hybridomas expressing cognate T cell receptors. Whether this phenomenon effectively involves the autophagic pathway or autophagy factors remains unknown. However, it could explain the protective immune response of MeV based-vaccines.

## 5. Conclusions

The infection of epithelial cells with MeV leads to a strong perturbation of the autophagy status of the cells. Attenuated forms of MeV rapidly trigger a transient phase of autophagy via the CD46-GOPC axis that promotes autophagosome formation through the BECN1-VPS34 complex. This phase declines without signs of negative regulation and appears thus to reflect cessation of induction. A few hours later, a second wave of autophagy that depends on viral replication is activated through interaction between MeV-C and the IRGM autophagy factor, and correlates with the accumulation of newly-synthesized viral proteins. Unlike the first wave, the second wave of autophagy is maintained over time and can be observed up to 48 h post infection. Such a sustained autophagic activity is, in fact, complex, since it also involves a pro-autophagy signal that originates from the cell-to-cell fusion events involved in syncytia formation. The virus takes advantage of this sustained wave of autophagy for its efficient replication, possibly though the constant supply of metabolites. In fact, MeV replication depends on a complete autophagy flux since it requires not only activation of the machinery involved in autophagosome formation (initiation-elongation), but also autophagy factors and events involved in the fusion of autophagosomes with endo-lysosomal vesicles (maturation). The anti-apoptotic effect associated with intense autophagic activity is likely to optimize the replication of MeV. Although beneficial to the virus at large, autophagy may be able to oppose MeV replication to some extent. For instance, silencing the autophagy receptor p62 promotes MeV replication, suggesting that it may target MeV components to degradation by autophagy. Intense autophagy may also promote the sensing of viral components by innate immune receptors within endosomes and activate type I IFN pathways. Finally, autophagy could facilitate the presentation of MeV epitopes on MHC class II molecules and, thus, promote the induction of CD4^+^ T cell responses within the adaptive immune system. In contrast with attenuated strains, virulent forms of MeV do not engage the CD46 receptor and, therefore, do not trigger the associated early wave of autophagy. Instead, due to amino-acid differences within the H protein, they interact with the CD150 receptor whose engagement appears not to cause detectable changes in the autophagy status of the host cells. The exclusive induction of a rapid and transient phase of autophagy by attenuated strains of MeV raises the issue of a possible relationship between the occurrence of such an early autophagy and the functional status of these strains. One could imagine that cellular events associated with early autophagy plays a role in the manifestation of the attenuated status, for instance through the rapid induction of anti-viral signaling pathways or via the early degradation of viral components. On the other hand, it is also possible that early autophagy is simply a consequence of the molecular characteristics of attenuated strains of MeV. Currently, such a question remains open. Despite our progress in understanding the crosstalk between autophagy and MeV infection, much remains to be learned: What are the roles of individual autophagy factors during MeV infection? What is the exact sensitivity of MeV components to autophagic degradation? To what extent can the autophagy machinery oppose viral replication? What role does autophagy play in MeV-infected immune cells? Answering such questions will help to clarify how MeV, and other pathogens, can manipulate autophagy and, in turn, how autophagy itself is regulated to achieve its various functions in order to maintain cell homeostasis.
